# Managing High Risk Pregnancy in Single Ventricle Physiology with Acquired von Willebrand Disease: A Case Report

**DOI:** 10.3390/reports8030157

**Published:** 2025-08-26

**Authors:** Yash Nagpal, Nisha Chachad, Paola Andrea Benito, Todd Stuart Roth, Joshua Saef

**Affiliations:** Adult Congenital Heart Disease Center, Memorial Cardiac and Vascular Institute, Joe DiMaggio Children’s Hospital, 3501 Johnson St., Hollywood, FL 33021, USA; ynagpal2022@health.fau.edu (Y.N.);

**Keywords:** Fontan circulation, acquired von Willebrand syndrome, single ventricle physiology, cardio-obstetrics, case report

## Abstract

**Background and Clinical Significance:** Left ventricular hypoplasia is often repaired surgically in sequence to a Fontan circulation, which is a physiologic state that presents unique challenges during pregnancy. Although women with Fontan physiology can achieve successful pregnancy outcomes, they remain at elevated risk for cardiac, thrombotic, and obstetric complications. **Case Presentation:** We describe a 38-year-old woman with Fontan physiology and acquired von Willebrand syndrome (AVWS) who was admitted at 23 weeks gestation for preterm premature rupture of membranes. The patient had history of prior classical cesarean delivery and two previous miscarriages. Her pregnancy was further complicated by abnormal placental vasculature and uterine arteriovenous malformation. Given her bleeding diathesis, hematology advised against anticoagulation or antiplatelet therapy, and she ultimately underwent a successful low transverse cesarean delivery under general anesthesia at 24 weeks. Postpartum hemorrhage was managed with clotting factor replacement and supportive care. **Conclusions:** This case illustrates how AVWS may mitigate thrombotic risk in Fontan physiology and how early activation of a cardio-obstetrics team can enable tailored planning. As more patients with complex congenital heart disease reach reproductive age, multidisciplinary coordination, shared infrastructure, and individualized birth plans will be essential to achieving optimal maternal–fetal outcomes.

## 1. Introduction and Clinical Significance

**Figure 1:**
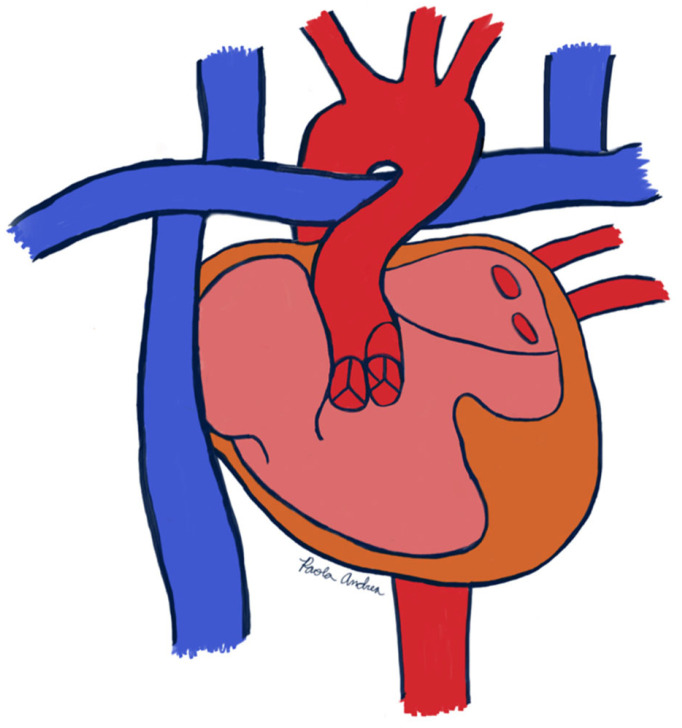
Depiction of patient’s anatomy.

Left heart hypoplasia (LHH) is a congenital underdevelopment of left-sided cardiac structures. The defect most notably involves hypoplasia of the left ventricular muscle mass, which becomes essentially nonfunctional, but often also includes underdevelopment of the mitral and aortic valves as well as the aortic arch [1] (Figure 1). Survival of neonates with LHH depends on patency of the ductus arteriosus and an atrial septal defect to maintain shunting, thereby allowing the right ventricle to supply both the pulmonary and systemic circuits [1].

LHH was previously considered a severely poor prognosis until the 1980s with the development of a three-step surgical intervention, which remains the current mainstay of treatment [1,2]. The first intervention, known as the Norwood procedure, is performed in the neonatal stage and involves widening of the atrial septal defect, reconnection of the right ventricle to the aorta, and creation of a shunt between the aorta and pulmonary artery to replace the ductus arteriosus [1,3]. The second intervention, the Glenn procedure, is performed between three and six months of age and includes detachment of the superior vena cava from the right atrium and re-anastomosis with a pulmonary artery [1,3]. This procedure thereby offers a more permanent solution to the shunt created by the Norwood procedure and reduces strain on the right ventricle [1]. Finally, the Fontan procedure is performed between two and three years of age [3]. During this intervention, the inferior vena cava also undergoes detachment from the right atrium and pulmonary artery re-anastomosis, and a small fenestration is created between the inferior vena cava and right atrium to prevent overload of the pulmonary circulation [1,3]. In doing so, the Fontan procedure decreases mixing of systemic venous return with pulmonary venous return, thus improving oxygen saturation [3]. The result of these procedures is a cardiovascular system known as single ventricle physiology. Within this mechanism, systemic venous return directly enters pulmonary circulation, and the right heart instead pumps blood into systemic arterial circulation, with complete bypass of the left ventricle. The prognosis of patients who successfully undergo all three procedures is variable. Patients with single ventricle physiology are at increased likelihood of developing heart failure, thromboembolic events due to excessive venous stasis, and arrhythmias due to cardiac scarring and remodeling that may lead to sudden cardiac death [3]. Obstruction of the aortic arch is another potential cause of morbidity and mortality among patients with a Fontan circulation system [1].

Even among healthy patients who have successful management of LHH due to functional single ventricle physiology, there are several management considerations in those who wish to undergo pregnancy [4]. Patients with Fontan circulation are at risk for both maternal and fetal adverse outcomes, including supraventricular arrhythmias, postpartum hemorrhage, fetal growth restriction, preterm labor, and even neonatal death [5,6]. During gestation, the maternal body transitions to a heightened metabolic state characterized by an expansion in plasma volume and elevation in heart rate [3,6]. These hemodynamic changes, accompanied by physiologic cardiac remodeling during pregnancy, further increase the risk of developing atrial arrhythmias in patients with Fontan circulation [3]. This increase in plasma volume corresponds to an increase in cardiac output and demand on the maternal circulation system, thus raising the patient’s risk of heart failure [7,8]. Furthermore, both single ventricle physiology and pregnancy contribute to a significantly prothrombotic state, made even more consequential by the fact that Fontan circulation involves development of a right-to-left shunt that creates potential for a cerebrovascular accident [3]. However, this risk is mitigated by functional closure of the Fontan fenestration [9]. In addition, studies examining maternal and fetal outcomes in previous pregnancies among patients with single ventricle physiology have demonstrated greater incidence of prematurity and fetal complications [10,11]. As a result, pregnancy in such patient populations is considered high risk for both mother and fetus, with up to a 70% risk of spontaneous abortion [8].

Another disorder influencing hemodynamic stability during pregnancy is von Willebrand disease (vWD), a bleeding disorder resulting from deficiency in von Willebrand Factor (vWF), which normally functions to promote platelet adhesion [12]. The disorder may be inherited or acquired due to an underlying medical problem. While acquired von Willebrand syndrome (AVWS) is commonly associated with lymphoproliferative and autoimmune disorders, patients with certain forms of congenital heart disease are also at increased risk of developing this condition [12]. In patients with single ventricle physiology, high-shear stress due to altered vessel pathways leads to increased proteolytic cleavage and therefore fewer high-molecular-weight multimers of vWF [12,13]. The combined impact of increased bleeding risk from AVWS as well as increased thromboembolic risk from both pregnancy and single ventricle physiology lends to an interesting discussion over the potential for coagulopathy in a patient experiencing all three conditions. Therefore, the objective of our case is to explore the hemodynamic considerations that were made in an obstetric patient with history of both acquired von Willebrand syndrome and Fontan circulation due to surgical correction of hypoplastic left heart syndrome.

Clinical Significance: Pregnancy in patients with Fontan physiology and acquired von Willebrand syndrome poses a uniquely complex hematologic and hemodynamic challenge due to the intersection of high thrombotic and bleeding risks, necessitating highly individualized, multidisciplinary care to optimize maternal and fetal outcomes.

## 2. Case Presentation

The patient under consideration is a 38-year-old female gravida 3, para 1 aborta 2 with past medical history of double outlet right ventricle with mitral atresia, severe left ventricular hypoplasia, D-malposed great arteries, aortic arch with mirror imaging branching, pulmonary stenosis, and bilateral superior vena cavae. She has had past correction via Norwood procedure, bilateral Glenn anastomosis, and non-fenestrated lateral tunnel Fontan procedure completed at 4 years of age (Figure 1). She has a history of stable mild-to-moderate systemic atrioventricular valve regurgitation with appropriate systolic function, which had been previously managed with angiotensin converting enzyme (ACE) inhibitor antihypertensives. Her previous abdominal ultrasound with elastography revealed elevated liver stiffness secondary to Fontan circulation, and past cardiopulmonary exercise testing demonstrated good functional capacity. Notably, the patient declined many of the diagnostic tests necessary for cardiac and hematologic evaluation; her main goal was for us to support her pregnancy from a medical standpoint.

Prior to the pregnancy discussed in this case, the patient had two previous pregnancies which both ended in intrauterine fetal demise due to preterm premature rupture of membranes with consequent umbilical cord prolapse at 26 weeks gestation and placenta previa at 16 weeks gestation, respectively. As a result, extensive consideration of the patient’s review of systems was performed during this pregnancy, particularly considering her additional risk factor of advanced maternal age. Aside from her history of infrequent episodes of palpitations, she also noted easy bruising and a previous adverse bleeding reaction to aspirin, leading to hematologic evaluation for acquired von Willebrand syndrome. The patient had a vWF antigen (vWF:Ag) of 264%, vWF ristocetin cofactor activity (vWF:RCo) of 140, vWF:RCo/vWF:Ag ratio of 0.53 (<0.6 supportive of qualitative defects) (Table 1). She also reported being told in her last pregnancy that she has an arteriovenous malformation of the uterus, which could impact perinatal outcomes. The patient received imaging via echocardiogram prior to conception, which confirmed her Fontan circulation system to be unobstructed with low flow velocity, indicating no volume overload. In addition, Holter monitoring revealed low supraventricular ectopic burden of 1.5% with only rare premature atrial contractions, indicating adequate electrical stability.

**Table 1:** Patient’s von Willebrand Panel.

Lab Component	Value
vWF antigen (vWF:Ag)	264%
Ristocetin cofactor activity (vWF:RCo)	140%
vWF:RCo/vWF:Ag ratio	0.53

During the patient’s initial prenatal visit at seven weeks gestation, she reported feeling well with no associated palpitations, shortness of breath, fluid retention, or presyncope. The patient was monitored throughout her pregnancy and was not found to have clinical signs (lack of abdominal distention, abdominal swelling, stable liver function tests) throughout her pregnancy. At 15 weeks gestation, she noted intermittent spotting, likely secondary to a subchorionic hematoma identified on prenatal ultrasonography but otherwise felt well. Echocardiography during the patient’s pregnancy revealed atrioventricular regurgitation, which was a point of concern to her cardiologist due to potential to prevent adequate flow through her Fontan circulation system and increase her postcapillary pulmonary pressures. The patient was admitted to the labor and delivery unit at 23 weeks gestation due to preterm premature rupture of membranes. She received antenatal corticosteroids as well as magnesium sulfate for fetal neuroprotection and remained hospitalized for an additional week prior to experiencing labor at 24 weeks gestation. Per hematology recommendation, she received no antiplatelet or anticoagulant medication due to suspected von Willebrand disease given her history of easy bruising and bleeding with aspirin use. Instead, her bleeding risk during delivery was managed with von Willebrand factor and clotting factor VIII replacement as well as tranexamic acid. She was also started on metoprolol to reduce cardiac workload during gestation by preventing an excessive increase in cardiac output and thereby decreasing stress forces on the heart.

Perioperatively, small bore femoral arterial and venous sheaths were placed as a precautionary measure in the event of excessive blood loss. The patient successfully delivered a female infant via low transverse cesarean section under general anesthesia; however, she still experienced postpartum hemorrhage, likely due to her history of acquired von Willebrand syndrome combined with suspicion for focal placenta accreta. This hemorrhage necessitated transfusion of two units of packed red blood cells and platelets intraoperatively. Following a brief postpartum admission to the cardiovascular intensive care unit for 48 h observation, the patient was de-escalated to the standard postpartum unit and was ultimately discharged with replacement vWF, Factor VIII, and tranexamic acid as needed due to elevated bleeding risk. Given that her child was admitted to the neonatal intensive care unit for respiratory failure secondary to prematurity, the patient requested expedited discharge. Postpartum follow-up data regarding the patient’s vWF status, bleeding profile, and need for ongoing factor replacement were not available, as patient did not complete these tests postpartum.

## 3. Discussion

Women with Fontan circulation are now surviving well into their reproductive years, making encounters like this increasingly common. Because these patients often receive care at multiple institutions over successive pregnancies, robust tracking systems and Adult Congenital Heart Disease (ACHD)-specific resources within shared electronic medical records are critical to avoid knowledge gaps and duplicate testing. Formal cardio-obstetrics teams that include adult congenital cardiology, maternal–fetal medicine, hematology, anesthesia, and neonatology should be activated at the first prenatal visit and maintained through the postpartum period. Such teams can standardize surveillance (e.g., serial echocardiography, iron indices, liver stiffness) and pre-empt the hemodynamic crises that drive maternal morbidity. A national registry capturing pregnancy outcomes in single ventricle physiology would further refine risk stratification and inform evidence-based guidelines. In short, system-level coordination will determine the successful management of future Fontan pregnancies. Additionally, although we were unable to obtain hematological data on the patient postpartum, we emphasize the need for close postnatal surveillance in patients with suspected AVWS, given the potential for rapid changes in hemostatic profile that may necessitate ongoing therapy.

This case highlights three actionable insights. First, acquired von Willebrand syndrome may paradoxically offset the formidable thrombotic risk of Fontan physiology by impairing platelet adhesion and aggregation, especially under the high-shear conditions common in the Fontan circuit, thereby blunting in situ thrombus propagation. Acquired von Willebrand syndrome alters standard management considerations for patients with Fontan circulation, for whom thromboprophylaxis is typically given in the form of either aspirin, warfarin, or direct oral anticoagulants [13,14]. Therefore, early recognition of the hematologic effect of AVWS is imperative, as withholding routine antiplatelet therapy can avoid compounding bleeding risk while modestly reducing platelet-mediated thrombosis.

Second, general anesthesia, with judicious avoidance of positive pressure ventilation, can be safely employed when neuraxial techniques are contraindicated by bleeding diathesis. Neuraxial anesthesia is associated with risk of hematoma formation at the insertion site, which has the potential to expand into potentially fatal vertebral canal hematoma [15]. This complication, although rare, is more likely in patients with pre-existing bleeding risk; therefore, given this patient’s history of AVWS, the decision was made to proceed with general anesthesia, which involves medication administration through a safer peripheral intravenous site. General anesthesia also allows for permissive hypotension, enabling the anesthesiology team to intentionally lower mean arterial pressure in order to reduce procedural blood loss [16].

Third, although trial of vaginal delivery is generally preferred in patients with adult congenital heart disease [17], elective low transverse cesarean delivery before labor onset mitigated the competing risks of uterine rupture after prior classical incision and of prolonged preload shifts during labor. In fact, a study of delivery trends from 2000 to 2018 found that pregnancies complicated by Fontan circulation were more likely to occur via cesarean section [18]. Taken together, these three points illustrate why a Fontan pregnancy is prototypical of complex ACHD care: seemingly contradictory hemodynamic and hematologic forces must be balanced in real time. When multidisciplinary teams craft individualized birth plans that consider the physiology of the patient, maternal, and neonatal outcomes can be optimized.

## 4. Conclusions

Pregnancy in single ventricle physiology remains high risk but is no longer prohibitive. Early engagement of a multidisciplinary cardio-obstetrics team, judicious anticoagulation strategy in the setting of acquired hemostatic disorders, and anesthesia plans tailored to Fontan hemodynamics collectively enabled a favorable outcome in this patient. As the Fontan population grows, scalable clinical care pathways and inter-institutional data sharing will be essential to reproduce this success. Continued collaboration between ACHD and obstetric specialists will transform what was once an anecdotal triumph into a predictable standard of care.

## Data Availability

The original data presented in this study are available on reasonable request from the corresponding author. The data are not publicly available due to privacy concerns.

## Abbreviations

LHH	Left Heart Hypoplasia
SVP	Single Ventricle Physiology
vWD	von Willebrand Disease
vWF	von Willebrand Factor
AVWS	Acquired von Willebrand syndrome
vWF:Ag	vWF antigen
vWF:RCo	vWF Ristocetin cofactory activity
ACE	Angiotensin Converting Enzyme
ACHD	Adult Congenital Heart Disease
